# Patient Commitment to Health (PACT-Health) in the Heart Failure Population: A Focus Group Study of an Active Communication Framework for Patient-Centered Health Behavior Change

**DOI:** 10.2196/12483

**Published:** 2019-08-06

**Authors:** Daniella Meeker, Jordan Goldberg, Katherine K Kim, Desi Peneva, Hugo De Oliveira Campos, Ross Maclean, Van Selby, Jason N Doctor

**Affiliations:** 1 Department of Preventive Medicine University of Southern California Los Angeles, CA United States; 2 stickK.com New York City, NY United States; 3 Betty Irene Moore School of Nursing University of California Davis Sacramento, CA United States; 4 Precision Health Economics Los Angeles, CA United States; 5 Kaiser Permanente Oakland, CA United States; 6 Cardiology Division, Department of Medicine University of California San Francisco San Francisco, CA United States; 7 Schaeffer Center for Health Policy and Economics University of Southern California Los Angeles, CA United States

**Keywords:** heart failure, behavioral economics, motivational interviewing

## Abstract

**Background:**

Over 6 million Americans have heart failure, and 1 in 8 deaths included heart failure as a contributing cause in 2016. Lifestyle changes and adherence to diet and exercise regimens are important in limiting disease progression. Health coaching and public commitment are two interactive communication strategies that may improve self-management of heart failure.

**Objective:**

This study aimed to conduct patient focus groups to gain insight into how best to implement health coaching and public commitment strategies within the heart failure population.

**Methods:**

Focus groups were conducted in two locations. We studied 2 patients in Oakland, California, and 5 patients in Los Angeles, California. Patients were referred by local cardiologists and had to have a diagnosis of chronic heart failure. We used a semistructured interview tool to explore several patient-centered themes including medication adherence, exercise habits, dietary habits, goals, accountability, and rewards. We coded focus group data using the a priori coding criteria for these domains.

**Results:**

Medication adherence barriers included regimen complexity, forgetfulness, and difficulty coping with side effects. Participants reported that they receive little instruction from care providers on appropriate exercise and dietary habits. They also reported personal and social obstacles to achieving these objectives. Participants were in favor of structured goal setting, use of online social networks, and financial rewards as a means of promoting health lifestyles. Peers were viewed as better motivating agents than family members.

**Conclusions:**

An active communication framework involving dissemination of diet- and exercise-related health information, structured goal setting, peer accountability, and financial rewards appears promising in the management of heart failure.

## Introduction

### Background

Cardiovascular disease is the leading cause of death in the United States and a source of rapidly increasing annual health expenditures [1]. Over 6 million Americans have heart failure, and 1 in 8 deaths included heart failure as a contributing cause in 2016 [2,3]. In addition, heart failure costs the nation an estimated US $32 billion each year and is projected to increase substantially over the next 20 years [1]. The Affordable Care Act targeted the economic burden of cardiovascular mortality by imposing financial penalties for cardiovascular-related hospital readmissions and offering financial incentives for numerous cardiovascular quality measures [4]. Given the significant burden of this disease and unmet need for improvement, a stronger focus on encouraging healthy behaviors among persons with and at risk for heart failure may be one of the best ways to improve patient outcomes.

Symptom burden and mortality risk are associated with behavioral factors among persons with or at risk for heart failure. Past studies have shown that moderate to high levels of physical activity are associated with a reduced risk of heart failure [5-7], whereas poor exercise capacity is associated with increased mortality risk in this population [8]. In addition, the American College of Cardiology and American Heart Association recommend low-sodium diets as a primary prevention for cardiovascular disease [9]. Some estimates suggest that as many as 1 in 20 heart failure admissions are precipitated by failure to follow dietary guidelines [10]. As physical activity and dietary habits are modifiable health-related behaviors that are directly associated with the risk of heart failure, the PAtient CommiTment to Health (PACT-Health) in the heart failure population study has high potential to improve health outcomes among persons with or at risk for heart failure by encouraging healthy behaviors through health coaching and public commitments.

Although evidence around managing heart failure has been established, guidelines are complex and often require behavioral change outside of the clinician visit. An important gap in evidence remains as to whether better communication of patients’ preferences and goals can improve adherence to plans developed with clinicians. Healthy lifestyle changes may improve clinical outcomes but require patients to engage in multiple health behaviors. Patient education as part of disease management programs delivered by multidisciplinary health care teams has been the primary communication and dissemination medium used to address disease self-management in this population. However, recent systematic reviews do not provide clear evidence for the effectiveness of this approach [11,12]. In general, the data indicate that self-care interventions can improve symptoms or prognosis [5] but often fail because of lack of persistence in their application [13].

With the premise that care planning requires thoughtful identification and communication of patient goals and mechanisms to motivate adherence to goals, we study the receptivity of patients to two research areas in behavior change: health coaching and public commitments. Health coaching is focused on evidenced-based approaches to setting goals and overcoming barriers that might prevent the achievement of goals, as well as in motivating lifestyle changes [14-16]. Health coaching is based on motivational interviewing, a procedure developed in clinical psychology to help people work through ambivalence about making changes in their life [17]. The simple idea is that patients do not enter into a dialogue ready to tackle changing their behavior. Giving direct advice often leads to defensiveness, resistance, and may exacerbate the behavior of concern. Health coaching asks the patients to articulate their own reasons for concern about their behavior and asks them for their own arguments for making a change. Strategy is matched to the patient’s readiness to change in brief educational modules. Information generation through health coaching is a patient-centered activity that requires the patient to generate all reasons for changes and concern over their behavior patterns and actions. By clearly developing these intentions, patients are able to more effectively represent their own interests and concerns when given the opportunity to discuss plans with clinicians.

Commitments follow readiness to change in health coaching. Since 2010, our group and others have been conducting studies showing that uptake of health-related behaviors is improved with the use of principles of psychology and economics to *nudge* persons toward making healthy choices [18-20]. We focus here on public commitment nudges—statements of dedication to a course of action that are visible to others [21-23]. People are more likely to follow through with their expressed intentions when that intention was framed as a commitment [24-26]. Public commitments have been shown to increase recycling [27-29], heighten participation in hotel towel reuse programs [30], boost monetary contributions to organizations serving the disabled [31], enhance the likelihood of voting in an upcoming election [32], reduce the prescription of inappropriate antibiotics [19], and lead dieters to lose more weight [33]. Public commitment is more effective than education as a tool to prompt greater personal motivation to perform a behavior [24,32,34]. Notably, public commitments are not forced by health providers nor formally negotiated agreements with another party but rather voluntary statements that strengthen dedication to an action and which later are identified with one’s values [35]. Commitment interventions are highly pragmatic. They minimize disruption of usual routines because they can be elicited in many different ways and take less than a minute to complete. Following through with a commitment makes a person’s actions consistent with their stated goals. Commitments then establish contingencies that enhance motivation to engage in self-management.

### Objective

This study aimed to conduct focus groups to gain insight into how best to implement health coaching and public commitment strategies within the heart failure population.

## Methods

Before commencement of focus group activities, we submitted a request to the Western Institutional Review Board for an exemption determination; the request was approved.

### Recruitment

Patients with chronic heart failure were recruited from a convenience sample through two cardiologists and two leaders of patient advocacy groups in Oakland, California, and Los Angeles, California. Participants were sourced during patient visits, through email and telephonic outreach, and via various social media posts. The primary obstacle to securing patient participation was physical difficulty (ie, health) or logistical difficulty (ie, can no longer drive or do not own a car) in leaving the home for an extended period of time. Patients were told that the discussion would last approximately 2 hours and would be recorded for transcription and follow-up analysis. Patients were given a US $100 cash incentive for participation in addition to a US $75 cash reimbursement for any travel expenses incurred.

### Interview Format

We used a semistructured interview tool (Multimedia Appendix 1) to explore several patient-centered themes: medication adherence, exercise habits, dietary habits, goals, accountability, and rewards. Following the interview portion, mockups of the Web-based user experience were presented to participants for their feedback. The mockups included both sample commitments (Multimedia Appendix 2) and sample rewards (Multimedia Appendix 3).

### Coding

A total of 2 reviewers coded the focus group data using codes developed *a priori* and evaluated responses from 2 reviews of each transcript. The Oakland transcript was 38 pages in length; the Los Angeles transcript was 44 pages in length. There were a total of 32 codes used (see Multimedia Appendix 4 for codebook). Published guidelines were followed to plan and report the study (Multimedia Appendix 5) [36].

The interviewers used several strategies to mitigate potential investigator bias. First, to address reflexivity, the interviewers discussed with other researchers on the team their assumptions, potential biases, and concerns before conducting the focus groups [37]. Second, the interviewers debriefed findings utilizing the transcripts with 2 peer researchers who did not attend the focus groups. Finally, quotes and findings were reviewed with 1 focus group participant to assess the accuracy of researchers’ interpretations of the findings.

## Results

### Sample Characteristics

We conducted two in-person focus groups. Table 1 displays the sample characteristics for participants from both groups.

The first focus group was held in Oakland, California, on March 16, 2015. There were 2 participants, both men with heart failure, ages 51 and 64 years. One of the men was Hispanic and both have lived for more than 5 years with heart failure. The intimate setting enabled us to essentially conduct two one-on-one interviews simultaneously, with both participants answering each question posed by the moderator. The second focus group was held in Los Angeles, California, on March 17, 2015. There were 5 participants of mixed sex, age, and race. The larger, open dynamic fostered more dialogue among participants, yielding more of a *conversational* tone than interview.

**Table 1:** Demographics of focus group participants.

Age (years)	Sex	Years with heart failure	Race, ethnicity	Location
51	Male	9	White, Hispanic	Oakland, California
64	Male	>5	White, non-Hispanic	Oakland, California
59	Female	<1	Other	Los Angeles, California
78	Female	4	White, Hispanic	Los Angeles, California
66	Female	17.5	White, non-Hispanic	Los Angeles, California
36	Male	1	White, non-Hispanic	Los Angeles, California
45	Female	9	White, non-Hispanic	Los Angeles, California

### Qualitative Findings

Participants readily shared their perspectives on living with heart failure. We began with a discussion of family history of heart disease and the moment of discovery of heart failure for each patient. A total of 1 participant was born with a heart condition and had corrective surgery at the age of 4 years and largely lived a normal childhood before experiencing heart failure episodes again at the ages of 26 and 34 years. Several reported having a pacemaker or a defibrillator. Several also shared a provocative event that began their lives living with heart failure. A total of 2 participants stated in no uncertain terms that their spouses left them as a result of the difficulties and stress of becoming a caregiver. Both participants have since found new significant others:

Nine years ago on my way to work before I even got to my car, I suffered a – sweating real bad, and couldn’t understand that. And by the time I got to my car, I passed out. When I woke up, I was in Kaiser Hospital. And that’s when Kaiser Hospital found out that I had CHF. I was angry. I was really angry.

I couldn’t get up the jet way [to the plane], and ended up in the local hospital. And, you know, one thing led to another, and the next thing you know, they slice me open when I got back to Austin, which has, by the way, fantastic doctors, and they put a device in me.

I was driving one day and I just got all sweaty and my heart was beating really fast and I went into a V-tach and went to the doctors and they hit me with the paddles and all that. Was there for three days and one of the doctors said, “We’re gonna install an ICD in you.” And I was like, “I don’t know.” It took some convincing but I got one.

#### Medication Adherence

The conversation continued with a discussion of medication compliance. Most felt that compliance was not a serious concern—participants understand the severity of their condition and the necessity for adherence. However, participants cited complexity of the regimen, forgetfulness, or difficulty adjusting to side effects as a reason for noncompliance at times:

Well, I’ve kind of gotten a schedule and I don’t necessarily have approval from my doctors because it is complicated and some of them say to take it at night, some say to take it with food, some say to take it without food.

It’s almost like the devil...which one do you want? You take it, you get so sick, and you don’t take it, you get sick because it’s in your system.

#### Current Exercise Habits

There was a spectrum of physical capacity to exercise, although most felt significant limitations in their abilities as a result of their condition. Exercise appeared largely limited to walking, biking, yoga, and short, light jogging. Los Angeles participants received very limited advice from their physicians on exercise regimens to follow. A total of 1 participant watched video tutorials for different exercise routines provided by her physician. The Oakland participants, on the contrary, both stated that they received clear advice from their physicians on exercise routines.

However, many felt uneasy about exercise because of fears and safety concerns that were neither endorsed nor discredited by their physicians. Many experienced tightness in their chest, rising heart rates, shortness of breath, and other forms of discomfort. A firm grasp of boundaries did not appear well understood. Outdoor temperatures (high heat) was cited as a big concern and an obstacle to exercise. In fact, one participant relocated from Texas to Northern California primarily for this reason.

Several participants mentioned that they make a habit of telling a loved one of their intentions to exercise before beginning, to make sure someone is aware in case there is a medical emergency that requires immediate attention as a result of exercising. Others indicated that even basic activities such as getting the mail were strenuous. Depression that stems from their condition was also cited as a hurdle to exercise:

...I find that walking, I can do it fine for a short time but then I get short of breath and I feel that tightness, so I just slow down.

My exercise is just daily life. There’s no regimen. And I always feel guilty about it but it doesn’t seem quite enough to push me into getting it done.

...if you don’t have that guidance, it’s really hard because the doctors sent me out of the hospital and said, “Okay, now you’ve got to exercise. You’ve got to eat right.” I was scared to death to exercise.

I was on all this medication and every time I exercised, I felt horrible. I thought I was going to die, or have a heart attack, or something.

I’m careful to keep it under – well, basically when I’m at the gym, I’m working at about just the heart rate of a hundred, and I’m shooting for that maximum, and leaving it there.

...I always let my wife know that I’m leaving, and she’s timing me to make sure. Because she knows that if it’s a certain time, she’ll come out looking for me. It’s just when I do walk, I get to the part that I have to stop a certain time to get some breath, and go back and walk; a certain time, catch a breath, or sit down because that’s basically my routine, and then I come back the same way.

I mean there is only a certain amount of things that the doctor can tell you because they don’t read inside your body. They can only go by the reports and how you feel and stuff, but it all depends on how my body takes it.

#### Current Dietary Habits

Similar to exercise habits, many participants received little guidance on diet from their physicians. One mentioned the Mediterranean diet as a specific recommendation from her doctor, but other participants received little specifics other than managing liquid intake and reducing sodium. Many participants claimed to still feel well informed on what to eat and what not to eat. Almost universally, participants cited reduction and strict limits on sodium intake to be a keystone practice in managing their health.

Difficulty sticking to a diet regimen was noted by several as a concern. Reasons cited included convenience of fast food, limited healthy options when dining out with friends and family, flavorful temptations of less healthy foods, and proximity to children’s or grandchildren’s unhealthy food. One participant mentioned boredom with the monotony of healthier foods. Many reiterated the importance of greater attention to healthy eating and a need to eat more fruits and vegetables specifically.

Another distraction to sticking with a diet plan is family. Participants cited family meals and gatherings as opportunities to eat unhealthy food; many found that family members are not as understanding or supportive as they would like them to be about their dietary needs:

I’ve been following the Mediterranean diet, a lot of grains, fruits, vegetables, and fish, and in general, just everything in moderation, so not a lot of one thing...But my diet’s not perfect. I drink too much coffee, and I eat chocolate sometimes, and I have young kids, too, and they want macaroni, and I sometimes eat their macaroni.

For me, I find that I’m not hungry, so I have to force myself to eat. I do eat vegetables. I’m not a meat eater.

I drink a lot of water. Your 64 ounces, that’s by 7:00 in the morning for me. I drink a lot of water.

[My Fitness Pal] lets me keep track of my liquids and my sodium. It’s awesome for tracking your sodium.

Well, bread is just so easy because you can put almond butter on it or when I cook fish, I cook extra so I can make a fish salad ’cause you can’t do tuna, canned tuna is too high in salt. But bread just makes portable meals and quick, easy, don’t have to fuss meals.

I eat a lot more salad now than I ever have. I also watch what I eat. Even when we go to the store and look at the box to decide – I was always wondering why my father was doing it, now I see why because you have to know how much salt you can take in. We’re the type of patients that we can’t have no salt. The doctor tells you, “No salt.”

Basically I eat a lot of fish, a lot of chicken and turkey. I very seldom have a piece of meat.

You wouldn’t believe how many times my nurse practitioner has been frustrated with me because the way I ate or the way I took my medications. Now, we understand each other.

I get contradictory recommendations because I also have type II diabetes. There are times when the heart doctor tells me this, and the diabetes doctor tells me that, and they don’t coincide.

#### Goals

Several participants noted the importance of goal setting in achieving new exercise, weight, or dietary milestones, but few have taken concrete actions to set goals. Most rely on resolve and willpower. One participant remarked that she is a “big list maker,” whereas two others noted the use of mobile apps to monitor and track fitness activity, eating habits, and glucose level.

A third participant mentioned that she has purchased a Fitbit, which she intends to use, but has not yet taken the time to set it up and begin using it. The remaining participants seemed largely unfamiliar with connected, wearable devices, but all expressed enthusiasm at the notion of what they can provide once it was explained to them. One participant expressed reluctance to use devices because it would serve as a reminder that he has a handicap and perhaps set off a depressive episode.

One participant noted his desire to get from “70 percent” to “80 or 90 percent,” while recognizing that 100 percent would be unlikely. He did not elaborate upon what it means to be 70 percent versus 90 percent. Another participant stated his intentions of doing more biking by increasing his mileage, not his pace, and has set concrete goals (in his mind) on how many miles he would like to do on a regular basis.

Several participants discussed goals more broadly. They shared their desire to spend active days with family and specifically grandchildren. Many also repeatedly stated their desire to be able to travel more regularly, or at all, and without the same degree of daily burden that their current condition imposes. One participant would like to get his band back together to play more regularly once he is able to confidently travel for longer periods of time:

I’m at 233 [pounds], and I want to go as far as making it to 180-200 [pounds]. That’s my goal right now.

I nudge – if I go four blocks, I try to get at least five in before I come in. So I move myself a little bit more and more to my goal.

I talk to myself a lot. I don’t seem to be very successful. Maybe I need to be talking to somebody else.

I write a lot of things down, but today I forgot my notebook, so I’m kind of bummed. That’s what I do. I usually just write everything down.

#### Motivation and Accountability

When discussing motivation and accountability, there was a vibrant conversation that covered many domains. A few common themes bore out. Participants appeared motivated to manage their health to be around for family, specifically children and grandchildren. Participants also noted on several occasions that at the end of the day, “it was on them” and that they should do it for themselves:

So here’s my family, I need to be there for them. I need to keep myself strong enough for them.

My grandkids...mean the world to me. I mean they know I can’t run around chasing them or anything like that, but I see them, and I see me. I only have one daughter, and she wonders if I took my medicines, or have I done this or done that. So she, in so many words, concerned also. So is my family…my mom, my dad, you know, my immediate family. It keeps me motivated.

My granddaughter turns ten next month and I really would like to see her at least graduate from high school but I’d like to see her graduate from college. She’s a pretty awesome little girl. But I don’t think I’m setting a lot of goals other than I want a quality of life.

The most commonly cited obstacle to feeling motivated to manage their health were bouts of depression. There was not a common theme with respect to triggers. Many participants had difficulty identifying triggers or consistency in their types of triggers. A total of 1 participant noted that at times if he noticed someone treating him too well, likely as a result of his condition, it would trigger a depressive episode. On the opposite end of the spectrum, the same participant noted that if someone is not treating him very well at all, it could trigger the same effect. Others said being reminded of their limitations in various forms can act as a trigger.

A total of 2 participants noted the ability that technology can play in motivation:

I just dig the numbers, the numbers that show up on the things that I’m logging, you know. That’s tangible enough for me to keep me going.

When the conversation progressed from motivation to accountability, there was a distinct shift in attitudes toward friends and family. Although many noted the importance they play in their desire to be healthy, many found family members to be a hindrance. Often family members are a source of temptation:

Because there are times when my family would love to force feed me barbecue, or any number of other things that can do me harm just because it’s a familiar pattern.

Although some noted that family members can be a basis for temptation, an underlying sentiment among many participants was simply that family members cannot be relied upon to hold them accountable in managing their daily habits. This did not appear to be a significant source of consternation. Rather, family members simply do not understand what sufferers are going through because it is a personal journey. Those who do not live with their conditions on a daily basis by definition cannot have an appreciation for it:

You know, like my brothers, they couldn’t understand.

Similar attitudes toward friends were pervasive but not to the same degree as family. Many participants shared the fact that they discuss and find comfort from confiding in contemporaries and would like to do so more. Spending time with friends and family can sometimes be seen as an escape.

At the moment, however, there is a similar issue of a lack of understanding with the condition. In addition, a couple of participants expressed their desire to not burden friends and family with their condition, or make the time they spend together depressing:

Yeah, and as much as my church friends, they try to be supportive, “Oh, you look so good and aren’t you glad that you’re all better now?” Sometimes I would say, “No. This is chronic and progressive.”

Though many participants felt friends did not understand their situation, they also noted that their friends often suffer from other ailments (more so than family members, particularly younger family members); as a result, there is some common ground for discussion and support:

Fortunately, I have many friends I’ve known for many, many years. We talk about anything, not just about the heart. They have their issues and we just support each other in that way.

With respect to tangible support and accountability, several participants discussed at length the importance and influence that their peer support groups provided. Specifically, a few participants were active members in online support groups on Facebook and other forums, and others were active in support groups that were local and had regular, in-person meetings. These participants were particularly vocal in their advocacy of the value of these networks and openly encouraged other participants who were not in such groups to join:

We’re not going to be normal, you know, 100 percent, but we need to hear from people who understand us.

The Facebook group...there is a lot of people that join right after they get sliced open and have a device put in... they’re all over the world. There are a lot of Canadians and English people.

Family loves you...But the group helps you.

Definitely the support group, ’cause they’re the peers who really get the struggle and they’re living it and they can relate. ’Cause friends who haven’t had congestive heart failure don’t understand.

One participant successfully formed a personal bond with her cardiac nurse practitioner and found her to be a powerful form of accountability and support. Another participant found that goals set by his doctor for his next visit was a strong form of accountability:

Well, I would have to say Peggy, who’s my cardiac nurse practitioner...she’s my biggest supporter when I have any kind of a health issue, or something I’m afraid of.

Another participant, who lives a somewhat remote life on a farm, eschewed the notion of sharing progress with anyone but herself. This is the same participant who uses My Fitness Pal, which she said keeps her focused and accountable:

No, I don’t have a caregiver and it’s not up to anybody else to hold me accountable. No, this is me and my life and I get to make the decisions.

The youngest participant, a male aged 36 years who was born with a heart condition, seemed content with not sharing his feelings with anyone, in part because of his gender and in part because of his age. He mentioned feeling isolated because his understanding is that few others his age have this condition. Two other participants sought to debunk the myth:

I guess, I live with my girlfriend, so I guess maybe her. I don’t really talk. I go to the doctor’s, I do my thing. I might come home and then I’ll talk about anything, and I don’t want to talk about my feelings, and I don’t talk to my friends about it, but I’ve also been like that for 36 years about anything in life that is troubling. I’m also a guy. We tend to do that all the time...It’s not a young person’s game, I guess, and so I don’t have a lot in common with people that are older. I guess that’s why I just kind of keep it to myself.

#### Rewards and Incentives

The conversation concluded with a discussion of what types of tangible rewards and incentives would motivate behavior change. There was a lively discussion in both groups, and there were many common ideas. When discussing rewards of significant value, travel as a reward was the most widely cited idea—from discounted airfare and other transportation, hotels, to activities at the destination.

An important insight is that the appeal of the reward was not merely the reward itself (ie, a fun trip) but the fact that traveling signifies a freedom and a loosening of restrictions that heart failure places on their lives. Rewards that speak to this would be quite powerful:

I would like to be able to go to places that I used to go.

I’ve been talking to the guys in the band about expanding our range, and buying a bus.

I would go to Vegas. I haven’t been to Vegas.

I’d go to Arizona and visit some friends.

When discussing lower cost rewards, an outing with friends or family to a restaurant was a popular theme:

I think I would go to a restaurant with my significant other, and enjoy the beach, and a nice meal – just a getaway, something simple.

Go out and eat and have a margarita.

Have a hamburger and a glass of wine.

I’d definitely have a beer.

Yeah, I think that would be the first thing is to go out to a nice dinner, not necessarily McDonald’s or In-N-Out, but to a nice place.

One male and one female participant suggested clothes shopping. This played into a similar notion that it was not just the reward itself (ie, new clothes) that would be motivating but the significance of what purchasing new clothes might represent (ie, weight loss and a renewed sense of self and freedom):

I enjoy doing that, and I don’t mind the shopping either because I can go out or buy a new outfit in a minute. But the thing is, I’m not ready. I’m not ready. And when I’m ready, that means the weight loss; I can be able to do that.

Two male participants suggested tickets to a sports game, specifically the Oakland Raiders in the Oakland focus group and the L.A. Dodgers in the Los Angeles focus group. Other participants suggested Japanese fountain pens that run approximately US $20.00, T-shirts, books, house plants, or simply anything fun that was not a simple cash reward:

I would spend it on something fun. My mom, she always gave us money for birthdays and Christmas and it was always, “You’re not to pay bills with this. You are to spend it on you.” And that always felt good.

Several participants asserted to their desire to spend the incentive on loved ones, in particular, on grandchildren:

I’d like to get my kid – her birthday is coming up, you know, so I thought I’d get her something nice for a change, maybe a nice purse or something. And then my grandkids, another toy, you know, they’re crazy about toys. So that’s basically it. Just to see smiles on their faces.

At this point in the focus group discussion, the mockups of the user experience were presented to the participants. Afterward, additional suggestions for goals and rewards were sought above and beyond what were shown on screen. Additional rewards suggestions included specific examples of activities for grandchildren, including museum tickets, movie tickets, aquarium passes (Long Beach and Monterey), Disneyland tickets, or an ice cream outing.

Other specific recommendations for rewards included Amazon gift cards, dinner cruises for 2, flowers, credits for cardiac rehab, and a high-end walker. One participant offered a conceptual suggestion of access to exclusive events, products, or services, one that money could not ordinarily purchase. Examples of this may include an invitation to a private dinner party, a local film screening, or literary reading:

I think the only thing that I would think of is it’s always nice to have access to something that you wouldn’t normally have access to, so something that you can get on your own? I don’t know what that would be, but some sort of exclusive behind-the-scenes kind of program or something.

#### Commitments

After the mockups were shown to the participants, many thought the list of commitments was fairly comprehensive and additional suggestions were tangential to the standard health and wellness goals offered on the platform mockups.

The participants suggested additional goals not shown included commitments to complete household tasks or hobbies such as laundry or putting together photograph albums. A few participants noted the ability to build a network of support through the site and how helpful that may prove to be:

Yeah. I would want it to be a network so that I can connect with others who have my same goal. The more you connect with others who have similar experiences, the more helpful it can be.

What I like about it is I find that I function better with accountability ’cause if I say something or do something, it’s important for me to complete it ’cause I feel better about it...if I’m accountable to someone and telling the truth, ’cause you can’t lie about your weight.

### Survey

At the conclusion of the focus group, participants were asked to complete a survey rating their interest in various rewards that would serve as an incentive to behavior change. Rewards fell into one of the following categories: health and fitness, luxury services, sports and leisure, home and family, or other items. Responses were recorded on a scale of 1 to 5, with a 1 denoting *I don’t want this at all*, a 3 denoting indifference, and a 5 denoting *I really want this*. All participants completed the survey.

Table 2 displays the medians and interquartile ranges for each reward, which are listed in order of most preferred to least preferred. Items with a median score of 5 include blood pressure or heart rate monitor, theater or symphony or opera tickets, and Fitbit, pedometer, or other activity tracker. Those with a median score of 2 or less included tension ropes and a round of golf at a local course.

**Table 2:** Preference for incentives.

Possible reward		Participant response (scale 1-5)^a^							Response, mean (SD)
		P1	P2	P3	P4	P5	P6	P7	
**Health and fitness**									
	Blood pressure or heart rate monitor	4	5	5	4	5	5	5	4.71 (0.49)
	Fitbit, pedometer, or other activity tracker	4	5	5	4	4	5	5	4.57 (0.53)
	Digital scale with body fat calculator	3	5	4	3	3	5	5	4.00 (1.00)
	Healthy dinner vouchers (4)	3	5	4	3	3	5	5	4.00 (1.00)
	Yoga mat	3	5	3	3	3	3	5	3.57 (0.98)
	Nike Plus (workout or run tracking device)	2	4	3	3	2	5	5	3.43 (1.27)
	Personal training session at gym	5	3	3	2	4	2	5	3.43 (1.27)
	Mountain or racing bike	4	5	4	1	1	5	4	3.43 (1.72)
	1-year subscription to *EatingWell* magazine	3	5	3	4	4	2	2	3.29 (1.11)
	1-month gym membership	3	3	3	5	3	3	2	3.14 (0.90)
	Track suit	2	5	3	3	3	2	4	3.14 (1.07)
	Flex ball	2	3	3	3	3	2	2	2.57 (0.53)
	Tension ropes	2	3	3	4	2	2	2	2.57 (0.79)
**Home and family**									
	House-cleaning service for 4 weeks	4	5	5	5	4	1	3	3.86 (1.46)
	Personal chef for dinner party for your family and friends	2	5	5	1	4	3	5	3.57 (1.62)
	Fruit or gift basket sent to your home	3	5	4	2	3	1	1	2.71 (1.50)
**Luxury services**									
	Theater or symphony or opera tickets (2)	5	4	5	4	5	2	5	4.29 (1.11)
	Wine tasting tour (2)	4	5	5	2	2	4	5	3.86 (1.35)
	1-hour massage	4	5	5	1	4	1	5	3.57 (1.81)
	Dinner cruise (2)	3	5	3	4	1	4	4	3.43 (1.27)
**Other items**									
	iPod Touch (16g)	4	5	4	4	4	5	5	4.43 (0.53)
	Gift card (eg, Target, Best Buy, Whole Foods, Nordstrom, and Sports Authority)	4	5	4	4	3	5	5	4.29 (0.76)
	Kindle or other electronic reader	4	5	4	5	3	5	1	3.86 (1.46)
	Recognition of your success in a spotlight section	3	5	4	1	3	4	5	3.57 (1.40)
	Compact digital camera	2	5	4	1	3	2	3	2.86 (1.35)
**Sports and leisure**									
	Movie tickets (2)	4	5	2	4	3	4	5	3.86 (1.07)
	Tickets to local sports event (eg, Angels, Giants, Lakers, Clippers, Kings) (2)	4	5	2	3	1	5	4	3.43 (1.51)
	Weekend spa passes (2)	5	4	5	1	2	1	5	3.29 (1.89)
	Disneyland park entries (4)	3	5	5	1	1	1	5	3.00 (2.00)
	Weekend organized bike trip (2)	4	4	2	1	1	3	4	2.71 (1.38)
	Round of golf at local course (2)	3	2	1	1	1	1	1	1.43 (0.79)

^a^Responses were recorded on a scale of 1 to 5, with a 1 denoting *I don’t want this at all*, a 3 denoting indifference, and a 5 denoting *I really want this*.

## Discussion

### Principal Findings

The focus groups yielded many insights relevant to the development of a patient-centered intervention for the management of heart failure. A few stood out as having particular importance. With regard to medication adherence, regimen complexity and logistics posed a problem for patients as did forgetfulness. These issues may present barriers to individuals’ readiness to consider behavior change, a prerequisite to making an actual commitment to change. For instance, a systematic review of past studies concluded that health coaching is one type of intervention that has produced positive effects on patients’ physiological, behavioral, and psychological conditions related to chronic illness in general [38] and heart failure specifically [39]. Commitment contracts are congruent with the health coaching process and can help reinforce action once an individual is ready to change. Commitments reinforce mindfulness through report reminders and creating layers of accountability, which will help with forgetfulness. The act of writing down a commitment (on paper or digitally), and the requisite details, will compel patients to put together a plan of action for compliance, which should help manage the complexity and logistical difficulty. Many participants cited the need to push through the first few months of a new regimen to either reduce or get acquainted with the side effects. Commitments that focus specifically on managing the side effects associated with the first few months of a new drug regimen can help improve adherence.

With regard to exercise, participants felt that heart failure imposes significant limitations on their ability to exercise. They also indicated that they receive little specific instruction from care providers. They were unsure of their own boundaries for safe exercise. Likewise, depression is often an obstacle to working out.

Commitment contracts, which address specific types of exercises most heart failure patients can perform, may be useful. Educating physicians on the need to improve communication about exercise is important. Developing a campaign that targets commitments around behaviors that address stress, anxiety, and depression management is also important.

As with exercise, participants reported receiving little instruction from providers with respect to diet. However, participants still felt well informed about what to eat and what not to eat. Limits on sodium intake and handling liquid intake are critical in managing their condition. Dining out with friends and family and temptations of unhealthy food nearby are primary drivers for noncompliance with a diet plan. Family members were not perceived as always understanding of the dietary restrictions that patients need to follow. Having users make a commitment to create a diet plan and share it with close friends and family so that they can better hold them accountable and not be a source of temptation may be an important strategy to improve diet among persons with heart failure.

Interestingly, few participants regularly practiced goal setting. Furthermore, there was little consistency in the goal setting process among the few that said they do practice goal setting. The use of technology, such as wearable devices, was of significant interest to the focus group participants. The development of commitments around device goals may be helpful. For instance, if a user has a commitment to walk 50,000 steps per week, a wearable device can validate this.

Family was considered a poor resource for structuring accountable commitments by the group. Patients often found that friends and family do not understand their conditions sufficiently to hold them accountable. Peer networks (both online and offline), particularly people who suffer from the same ailments, are very powerful centers of influence and accountability. They are seen as partners in their journey toward managing their disease. Use of a peer referee may facilitate greater and more intimate interaction with both online and real-world networks. As a lack of understanding and responsiveness from loved ones is apparent in the responses, creating a framework for better educating loved ones about heart failure in an effort to boost their ability to hold their loved ones accountable may also be needed. Similarly, no participant mentioned favoring personal accountability, which may suggest that a patient’s motivation to manage their health is at odds with the helplessness they feel in managing their condition. Encouraging autonomy and personal accountability may facilitate better health management.

Finally, participants were very amenable to leveraging incentives for motivation. Expectations that rewards will encourage better health are fair and justified. Careful development of incentive choice sets seems important. Particularly, there will be a need to develop material rewards that speak to the diverse tastes of individuals with heart failure.

### Study Limitations

There are several limitations to this study. Focus group research is qualitative in nature, often providing insufficient insight into the general pattern of behavior to develop of a strong set of intuition, which can be used to refine concepts or assist in the development of new research ideas. As in the case of any qualitative research, the findings presented herein may have been influenced by the context of the focus group. Finally, our findings are limited given the small sample size. We were unable to recruit a greater number of participants because many of the referred patients were too ill or did not have the physical ability to travel to the focus group locations owing to their condition. We hope future work in the chronic heart failure population can address this limitation.

### Conclusions

In summary, we evaluated patients’ overall outlook concerning their heart condition. We asked about the steps that participants have taken (eg, medication adherence, exercise, and diet) and the specific goals they have set to manage their condition. We studied what and who motivates their behavior. Overall, we found that medication regimens were complex and involved adverse side effects. We also found that providers did not provide exercise and diet recommendations. There was an interest in goal setting but a lack of practice in doing so. Participants favored peer accountability for goals rather than family member accountability. Finally, financial incentives were viewed favorably as a means to motivate behavior change.

## Appendix

Multimedia Appendix 1 Interview tool.

Multimedia Appendix 2 Sample commitments.

Multimedia Appendix 3 Sample rewards.

Multimedia Appendix 4 Codebook.

Multimedia Appendix 5 Consolidated Criteria for Reporting Qualitative Studies (COREQ): 32-item Checklist.
