# Supplementary material for: Patient Commitment to Health (PACT-Health) in the Heart Failure Population: A Focus Group Study of an Active Communication Framework for Patient-Centered Health Behavior Change
Source: J Med Internet Res. 2019 Aug 6;21(8):e12483. doi: 10.2196/12483 (PMC6701162; doi:10.2196/12483)
Supplement: Multimedia Appendix 1 [file jmir_v21i8e12483_app1.docx]

**Appendix 1. Interview Tool**

**Background (20 minutes) – Current Attitudes and Actions**

1. When it comes to your health—what is the first thing that comes to mind?
2. How do you currently manage your health? Are there certain things or activities in which you engage?
3. What are some of the things you’d like to do to better manage your health that you don’t currently do?
4. What are some of the reasons you don’t do these things you’ve just named?
5. Have you contemplated surgery? How would the possibility of needing surgery influence your actions?

**Motivation (20 minutes) – Rewards and Incentives**

We are going to continue the discussion by having you think about motivation. People are motivated by different things; what are some things that motivate you in your life?

**Additional prompts if necessary:**

1. What types of incentives appeal to you?
   1. If someone asked you to fill out a questionnaire that would take 10 minutes, what type of reward would make you more likely to agree?
      1. If needed for prompt: e.g., a t-shirt, coffee mug, iTunes gift card, movie ticket?
2. When you need to do something you are not looking forward to, how do you convince yourself to do it?

## **Goals**

I would like to shift the conversation to setting goals for yourself.

1. What are some goals you’ve set for yourself?
2. What are the challenges you’ve faced in achieving those goals?
3. Are there any health related goals you’ve set?
4. Are there any recommendations your doctor has made that you think you might want to set a goal around? Or that you **should** set a goal around?
5. What are some of the most challenging recommendations your doctor has suggested?
6. What have you done to try to make the changes your doctor suggested?

## **Referee and Supporter Section**

1. Whose opinions about what you do matter most? What motivates you to achieve your goals in life?
   1. Has this answer changed over time?
2. Who in your life pushes you to want to achieve a goal? Who would be happiest to see you succeed?
3. When managing your health, who is motivating you?
4. When you think about managing your health, how much of your motivation is driven by doing it for yourself or doing it for others around you?
   1. If some of the motivation is others, who are those people and why?
5. Does anyone come with you to visit the doctor?
6. Do you tell anyone about the visit after? If so, do you ever leave anything out?

**More on incentives:**

1. What do you see as the personal benefits to taking care of your health?

## **Experiences**

1. What sort of activities would you want to do with these people to celebrate your accomplished goals?
   1. If needed for prompt: e.g., amusement park, bowling, similar activities?
2. OR REPHRASE: What sort of activities do you do to celebrate small, daily/weekly accomplishments with your family and friends? How does this change for longer term, more life changing accomplishments?
   1. Immediate gratification (buy a swim suit with your girlfriends or go to a low cost event today like dinner and bowling) vs. deferred gratification (plan a cruise to show off weight loss with friends or plan a family trip)
3. What other kinds of rewards would be motivating and fun to earn for being successful in the health goals we have been sharing?
   1. If needed for prompt: e.g., kindle, digital camera, other consumer electronics, gift cards?

**If not mentioned earlier:**

1. Is being recognized in front of your peers motivating?
2. What specific activities do you like to do as a reward?
