# Supplementary material for: Patient Commitment to Health (PACT-Health) in the Heart Failure Population: A Focus Group Study of an Active Communication Framework for Patient-Centered Health Behavior Change
Source: J Med Internet Res. 2019 Aug 6;21(8):e12483. doi: 10.2196/12483 (PMC6701162; doi:10.2196/12483)
Supplement: Multimedia Appendix 2 [file jmir_v21i8e12483_app2.docx]

# **Appendix 2. Sample Commitments shown During Focus Group**

1. My Regimen
   1. Follow My Prescription Regimen
   2. Complete a Heart Failure Self-Care Seminar
   3. Complete My Nightly CPAP
   4. Sleep at Least 8 Hours
   5. Go to Heart Failure Support Group
2. Healthy Me
   1. Maintain Weight
   2. Lose Weight
   3. Lower BMI
   4. Quit Smoking
   5. Sober Challenge
3. Physical Activity
   1. Hit the Gym
   2. Exercise Regularly
   3. Walk 15 Miles Every Week
   4. Go to Spin Class 4 Times a Week
   5. Take the Stairs Instead of Elevator
4. Nutrition
   1. Lower My Sodium Intake
   2. Drink 8 Glasses of Water Everyday
   3. Eat More Whole-Grain Foods
   4. Have 5-9 Servings of Fresh Fruits and Vegetables per Day
   5. Take an Organic Cooking Course
   6. Limit Processed and Fried Food Consumption
5. Personal Relationships
   1. Have Family Dinners on Sunday
   2. Take Evening Walks with My Spouse
   3. Screen-Free Time Everyday
   4. Visit a Friend or Family Member Every Week
