# Supplementary material for: Patient Commitment to Health (PACT-Health) in the Heart Failure Population: A Focus Group Study of an Active Communication Framework for Patient-Centered Health Behavior Change
Source: J Med Internet Res. 2019 Aug 6;21(8):e12483. doi: 10.2196/12483 (PMC6701162; doi:10.2196/12483)
Supplement: Multimedia Appendix 3 [file jmir_v21i8e12483_app3.docx]

# **Appendix 3. Sample Rewards Shown During Focus Group**

1. Redeemable Prizes
   1. 3 Month Membership to 24-Hour Fitness
   2. $25 Gift Card to Their Choice of Retailer
   3. 4 AMC Theater Movie Passes
   4. Fitbit Zip or Fitbit One Device
   5. Escali Glass Body Fat, Water, and Muscle Mass Scale
   6. 1-Hour Massage at The Massage Co.
2. Raffles
   1. Mountain Bike
   2. iPad (16 GB) with Retina Display
   3. 2 Tickets to a Choice of Sports Team Game
   4. 4 Tickets to Disneyland California Adventure Theme Park
