# Supplementary material for: Patient Commitment to Health (PACT-Health) in the Heart Failure Population: A Focus Group Study of an Active Communication Framework for Patient-Centered Health Behavior Change
Source: J Med Internet Res. 2019 Aug 6;21(8):e12483. doi: 10.2196/12483 (PMC6701162; doi:10.2196/12483)
Supplement: Multimedia Appendix 4 [file jmir_v21i8e12483_app4.docx]

**Appendix 4. Codebook Used to Code Focus Group Data**

1. **Death of loved ones:** instances where participants reveal the death of family members or friends associated to a medical cause

2. **Dependent because of CHF:** instances where participants discuss becoming dependent on others because of CHF

3. **Depression:** instances where depression is brought up or insinuated

4. **Diagnosis:** moments where participants talk about their diagnosis and how they got to that diagnosis

5. **Family medical history:** any instance where participants share family medical history, whether positive or negative

6. **Family support:** any statement of support coming from family members

7. **Friends support:** any statement of support coming from friends

8. **Future with family:** any instance of longing to survive long enough to see certain moments in the lives of family members

9. **Good habits:** routine activities that participants have picked up to keep a healthier lifestyle since their diagnosis

10. **Health goals:** habits or activities that participants would like to pick up to live healthier lifestyles

11. **Healthy progress:** any instances that reveal strides in the right direction by the participants

12. **Healthy routine deterrents:** any people, actions, instances, or natural forces that could derail healthy lifestyle choices for the participants

13. **Interactions with physicians:** any instances where a participant seeks out medical advice or is told to start a certain regime

14. **Life consequences (broad):** any effects caused by the fact that these patients now have to live with CHF

15. **Medical consequences:** any moments where participants relay events that happened to them as a result of being on certain medications

16. **Medical education:** any instances where participants retell of having to do their own research or ask for medical advice directly

17. **Medical emergencies since diagnosis:** any instances where participants recount medical emergencies since diagnosis

18. **Medications taken:** any moments where participants share their medication regimes

19. **Mobile application:** instances where participants mention using mobile apps to help them achieve a healthier lifestyle

20. **Motivation:** any instances where participants share what currently or could motivate them to achieve their health related goals

21. **Negative feelings towards CHF:** any instances where participants express how CHF have negatively affected their lives

22. **Old bad habits:** any routines that participants had before diagnosis, which they know are unhealthy, that they continue to practice

23. **Positive feelings towards CHF:** any instance where the participants sees the silver lining or an optimistic future while living with CHF

24. **Potential commitment contract:** different instances where an idea for a commitment contract can be built from

25. **Recovery:** any stories about the road to recovery, usually from the first instance that resulted in diagnosis

26. **Rewards or experiences for success:** potential prizes or experiences that could be used to motivate participants to achieve the goals they set out to make

27. **stickK:** any comments related to stickK made by the participants, or how stickK could fit into what they are searching for in a program

28. **Support group:** any broad indication of participating in a support group, whether in person or online, by the participants

29. **Technology:** applicable mentions of how technology, whether through their pacemakers or tablets or health apps, affects the everyday lives of participants

30. **Triggers:** any instances where participants note a certain action or limitation that can cause them to be upset, relating to the fact that they have CHF

31. **Weight gain:** any notes of how much weight has been gained since diagnosis by the participants

32. **Weight loss:** any comments about weight loss that has already been achieved, or that is a goal weight for them to achieve
