# Supplementary material for: Patient Commitment to Health (PACT-Health) in the Heart Failure Population: A Focus Group Study of an Active Communication Framework for Patient-Centered Health Behavior Change
Source: J Med Internet Res. 2019 Aug 6;21(8):e12483. doi: 10.2196/12483 (PMC6701162; doi:10.2196/12483)
Supplement: Multimedia Appendix 5 [file jmir_v21i8e12483_app5.docx]

**Appendix 5. Consolidated Criteria for Reporting Qualitative Studies (COREQ): 32-item Checklist**

| No. Item | Guide questions/description | Reported on  Page No. |
| --- | --- | --- |
| RESEARCH TEAM AND REFLEXIVITY | |  |
| Personal characteristics | |  |
| 1. Interviewer/ facilitator | Which author/s conducted the interview or focus group? | Title page |
| 2. Credentials | What were the researcher's credentials? *E.g. PhD, MD* | Title page |
| 3. Occupation | What was their occupation at the time of the study? | Title page |
| 4. Gender | Was the researcher male or female? | Title page |
| 5. Experience and training | What experience or training did the researcher have? | Title page |
| Relationship with participants | |  |
| 6. Relationship established | Was a relationship established prior to study commencement? | Methods |
| 7. Participant knowledge of the interviewer | What did the participants know about the researcher? e*.g. personal goals, reasons for doing the research* | Participant information sheet and Consent Form |
| 8. Interviewer characteristics | What characteristics were reported about the interviewer/facilitator? e.g. *Bias, assumptions, reasons and interests in the research topic* | Methods |
| STUDY DESIGN | |  |
| Theoretical framework | |  |
| 9. Methodological orientation and theory | What methodological orientation was stated to underpin the study? *e.g. grounded theory, discourse analysis, ethnography, phenomenology, content analysis* | Methods |
| Participant selection | |  |
| 10. Sampling | How were participants selected? *e.g. purposive, convenience, consecutive, snowball* | Methods |
| 11. Method of approach | How were participants approached? e*.g. face-to-face, telephone, mail, email* | Methods |
| 12. Sample size | How many participants were in the study? | Results/Sample characteristics |
| 13. Non-participation | How many people refused to participate or dropped out? Reasons? | Methods |
| Setting | |  |
| 14. Setting of data collection | Where was the data collected? e*.g. home, clinic, workplace* | Hotel conference room setting |
| 15. Presence of non-participants | Was anyone else present besides the participants and researchers? | No |
| 16. Description of sample | What are the important characteristics of the sample? *e.g. demographic data, date* | Results/Sample characteristics |
| Data collection | |  |
| 17. Interview guide | Were questions, prompts, guides provided by the authors? Was it pilot tested? | Appendix A |
| 18. Repeat interviews | Were repeat interviews carried out? If yes, how many? | No |
| 19. Audio/visual recording | Did the research use audio or visual recording to collect the data? | Methods |
| 20. Field notes | Were field notes made during and/or after the interview or focus group? | Yes |
| 21. Duration | What was the duration of the interviews or focus group? | Methods |
| 22. Data saturation | Was data saturation discussed? | No |
| 23. Transcripts returned | Were transcripts returned to participants for comment and/or correction? | Methods |
| ANALYSIS AND FINDINGS | |  |
| Data analysis | |  |
| 24. Number of data coders | How many data coders coded the data? | Methods |
| 25. Description of the coding tree | Did authors provide a description of the coding tree? | No |
| 26. Derivation of themes | Were themes identified in advance or derived from the data? | Methods |
| 27. Software | What software, if applicable, was used to manage the data? | ATLAS.ti |
| 28. Participant checking | Did participants provide feedback on the findings? | Methods |
| Reporting | |  |
| 29 Quotations presented | Were participant quotations presented to illustrate the themes / findings? Was each quotation identified? e*.g. participant number* | Results |
| 30. Data and findings consistent | Was there consistency between the data presented and the findings? | Yes.  Results |
| 31. Clarity of major themes | Were major themes clearly presented in the findings? | Yes.  Results |
| 32. Clarity of minor themes | Is there a description of diverse cases or discussion of minor themes? | Results |

Note: Developed from: Tong A, Sainsbury P, Craig J. Consolidated criteria for reporting qualitative research (COREQ): a 32-item checklist for interviews and focus groups. International Journal for Quality in Health Care. 2007;19(6):349-57.
